# Ultra-processed foods and cardiometabolic risk: from evidence to policy

**DOI:** 10.1038/s44324-026-00116-2

**Published:** 2026-06-02

**Authors:** Hayley M. O’Neill

**Affiliations:** Faculty of Health Sciences and Medicine, Bond University, Robina, QLD Australia

**Keywords:** Diseases, Endocrinology, Health care, Risk factors

## Abstract

*Ultra-processed foods (UPFs)* dominate diets in high-income countries and pose health risks beyond nutrient composition. Controlled trials show UPF-rich diets increase energy intake and weight gain. Mechanisms include high energy density, disrupted food matrices, faster eating rates, additives affecting gut, and hyper-palatable formulations. Observational evidence associates higher UPF intake with obesity, cardiovascular disease, type 2 diabetes and all-cause mortality. *This Comment outlines evidence and policy strategies to reduce UPF exposure.*

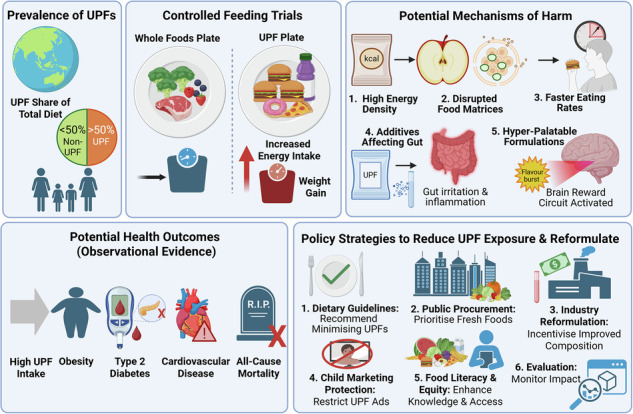

## Introduction

Ultra-processed foods (UPFs) account for a substantial share of daily energy in the US, UK, Canada, Australia, and across Europe, with health impacts that extend beyond nutrient content^1,2^. Although the term ‘UPF’ lacks universal definition, NOVA classification system is most widely used, distinguishing UPFs by their processing and use of additives^3^. UPFs are industrial formulations composed of fractionated ingredients (e.g. refined starches, hydrogenated oils, protein isolates, high-fructose corn syrup) combined with cosmetic additives (e.g. emulsifiers, flavours, colours, preservatives, sweeteners) and subjected to industrial processes such as extrusion and pre-frying^3^. These techniques enhance palatability, convenience, affordability, and shelf life, exemplified by products like breakfast cereals, sugar-sweetened beverages (SSBs), packaged bakery items, mass-produced bread, instant noodles, and ready-made meals^3^. While processing has improved preservation and access, growing evidence suggests that the degree and purpose of processing may independently disrupt appetite regulation, gut microbiota, and metabolic signalling contributing to elevated elevate cardiometabolic risk^2,4,5^. As global UPF sales continue to rise, understanding these implications is critical for effective nutrition policy^2,3^.

## Evidence at a glance

### Observational syntheses

Overviews of systematic reviews and meta-analyses generally report positive associations between higher UPF intake and obesity, type 2 diabetes, cardiovascular disease, and all-cause mortality; certainty ranges from weak to moderate depending on design and bias assessment^2,6^. Some reviews (e.g. Monteiro 2025^2^) have not applied standardised frameworks (e.g. GRADE [Grading of Recommendations Assessment, Development and Evaluation] to formally assess all of the evidence reviewed or ROBINS-E [Risk of Bias In Non-randomised Studies of Exposure] despite being more appropriate for dietary exposure studies than commonly used Newcastle-Ottawa Scale); limiting transparent evaluation and risking overstatement of findings from predominantly observational studies^7^.

### Burden-of-Proof reappraisal

**Fig. 1:**
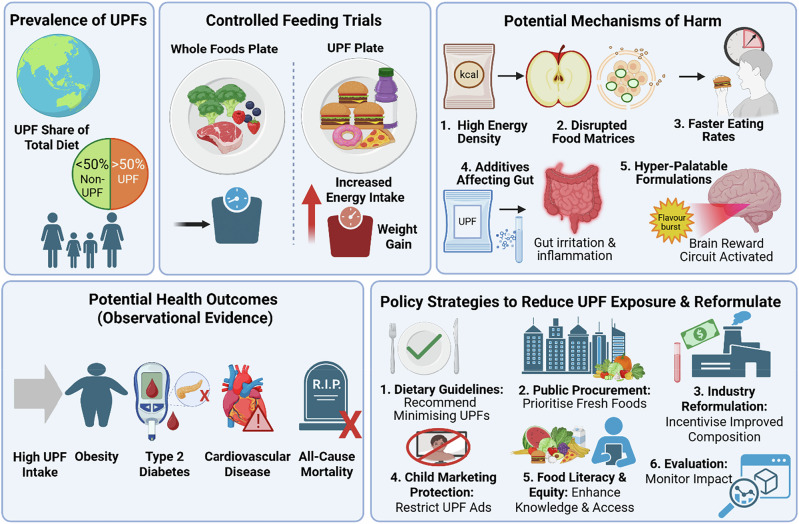
Potential health risks and policy strategies associated with ultra-processed foods (UPFs). In many high-income countries (e.g. USA, Canada, UK, and Australia), UPFs contribute more than 40–50% of total dietary energy intake. Examples of UPFs include soft drinks, breakfast cereals, bakery items, ready-to-eat/heat pre-prepared meals e.g. burgers, pizza. Evidence from controlled feeding trials and observational studies indicates that UPFs pose health risks beyond nutrient composition alone. High energy density and disrupted food matrices resulting from industrial processing promote faster eating rates, while hyperpalatable formulations rich in sugar, salt, and fat can encourage excess energy intake and weight gain. Certain food additives, including emulsifiers, may adversely affect gut health. High UPF exposure may be associated with increased risk of obesity, type 2 diabetes, cardiovascular disease, and all-cause mortality. Large-scale dietary change remains challenging without aligned incentives across industry, government, and households. Potential policy strategies to reduce UPF exposure include dietary guidelines recommending minimisation of UPFs; public procurement policies prioritising minimally processed foods; incentives for industry reformulation; restrictions on marketing to children; initiatives to improve food literacy and equitable access; and ongoing evaluation to monitor policy impact. Created in BioRender. O’Neill, H. (2026) https://BioRender.com/hh4d6v5.

A recent Burden-of-Proof study examined health risks of processed meats, SSBs, and trans fats^8^. This approach uses Bayesian meta-regression to model dose-response relationships relative to zero intake, establishing conservative risk estimates while accounting for between-study heterogeneity. These findings indicate that consumption of processed meat (upto 57 g/day) is associated with 11% higher type 2 diabetes risk and 7% higher colorectal cancer risk; SSBs (upto 390 g/day) with 8% higher type 2 diabetes risk and 2% higher ischaemic heart disease (IHD) risk; and trans fats (upto 2.5% of daily energy) with >3% higher IHD risk^8^. Most ratings were ‘two-star’, indicating weak or inconsistent evidence, yet continued limitation remains prudent and aligns with current guidance (e.g. free sugars <25 g/day [~6 tsp], SSBs <1 serving/week [~200–355 mL], TFAs <2% energy)^8,9^. Important limitations include potential residual confounding from unmeasured variables (e.g. total energy intake) and reliance on self-reported dietary intake despite statistical adjustment^8,10^ (Fig. 1).

### Select randomised controlled trials (RCTs)

UPF feeding trials remain limited (*n* ≈ 10–55; duration 2 days–8 weeks) but converge on unfavourable acute effects on energy intake and weight^11–16^. In Hall’s landmark inpatient crossover RCT (*n* = 20 overweight; 2 weeks), UPF diets yielded ~500 kcal/day greater intake and ~0.9 kg weight gain versus unprocessed diets, despite matched calories, macronutrients, sugar, sodium, and fibre^11^. The UPDATE trial (crossover RCT; *n* = 55 overweight/obesity; 8 weeks) extended these findings under UK Eatwell Guide conditions: ad libitum minimally processed food (MPF) patterns produced greater weight loss (−2% vs. −1%) and improvements in LDL-cholesterol, apoB, and HbA1c versus UPF patterns, under matched nutrient targets^17^. These trials provide high-quality data on short-term responses, yet are limited by: small sample sizes and durations, lack of formal risk of bias assessment, specific populations and settings (overweight/obesity, inpatient vs free living, affecting generalisability), challenges in dietary assessment methods and adherence monitoring, and logistical and financial demands of all-food provision, collectively reducing statistical power to detect modest clinical effects^11,17^ (Table 1).

**Table 1:** Select randomised controlled trials examining ultra-processed food (UPF) exposure and energy intake, body weight or eating rate outcomes

Author (year), country	Population/Inclusion criteria	*N*; Age; sex (% female), BMI	Intervention	Duration	Comparator/control	Outcome/key measures	UPF Exposure (NOVA)	Key Findings
Dicken et al.^17,64^ UPDATE Trial UK (England)	Adults with overweight/obesity	*N* = 55, 43 y; 90.9% F; BMI 33	Ad libitum MPF diet (UK Eatwell Guide-aligned)	2 × 8 wk	Ad libitum UPF diet (UK Eatwell Guide-aligned)	Body weight change; fat mass; DXA; cardiometabolic biomarkers	Monteiro et al. (NOVA)^3^; MPF vs UPF	MPF led to ↑ weight (−1.01) and fat mass (−0.98) loss. No differences in blood biomarkers between diets.
Forde et al. ^19^ Netherlands	Healthy adults	*N* = 41; 27 y; 49% F; BMI 23	Ad libitum UPF fast eating rate diet (soft textures)	2 × 14 d	UPF Slow eating‑rate diet (hard/chewy; matched)	Body weight; DXA; 3-day food diary and FFQ (dietary intake); eating rate (g/min).	Monteiro et al. (NOVA)^3^. Both arms >90% energy NOVA 4 (matched between arms)	Slower eating rate ↓energy intake (−369 kcal/d) on UPF Slow-ER compared with UPF Fast-ER diet. No difference in body weight between diets.
Hägele et al. ^13^ Germany	Healthy young adults	*N* = 24 (21 ITT); 25.7 y; 57% F; BMI 24	Ab libitum high‑protein low-CHO UPF diet (30% protein)	2 × 54 h	Normal‑protein normal‑CHO UPF diet (13% protein, 46% CHO); matched for energy, fat, fibre and palatability	Energy intake; EE; whole-room indirect calorimeter; weighed food intake; gut hormone assays; gastric emptying; appetite VAS	Monteiro et al. (NOVA)^3^; >84% energy from NOVA 4 (both arms)	High‑protein UPF ↓ energy intake slightly (−196 ± 396 kcal/ d), and increased EE (+128 ± 98 kcal/d), but did not prevent net overeating.
Hall et al. ^11^ USA	Healthy weight‑stable adults	*N* = 20; 31.2 y; 50%F; BMI 27	Ad libitum UPF diet (inpatient)	2 × 14 d	Ad libitum unprocessed diet (88% energy from NOVA 1–2); matched for energy, macronutrients, sugar, fibre, sodium	Ad libitum energy intake; weighed food intake; body weight change; DXA; DLW; respiratory chamber	Monteiro et al. 2018 (NOVA groups 1–4)^65^; UPF 81% energy vs unprocessed (NOVA 1–2)	UPF diet ↑ intake (+508 kcal/d) and (+0.9 kg) weight gain vs loss (−0.9 kg) on unprocessed diet
Hamano et al. ^14^ Japan	Overweight/obese males; no diabetes, hypertension, or chronic conditions	*N* = 9; 30 y; 0% F; BMI 27	Ad libitum UPF diet; all meals provided by the hospital	2 × 7 d	Ad libitum non‑UPF diet; matched for total energy and macronutrients	Body weight; weighed food intake; chewing frequency (video‑based counting)	Monteiro et al. 2018 (NOVA)^65^; UPF ≥ 50% energy NOVA 4; dietitian‑designed menus	UPF↑ energy intake (+814 kcal/day) and weight (+1.1 kg); ↓ chewing frequency per calorie
Teo et al. ^15^ Singapore	Healthy‑weight adults	*N* = 50; 24 y; 52%F; BMI 21	Four ad libitum lunch meals crossing texture (soft/hard) and processing (UPF/MPF): soft UPF, hard UPF, soft MPF, hard MPF	Four single‑meal sessions	Hard minimally processed meal (HMP) (reference)	Energy intake; eating rate; post‑meal satiety. Weighed food intake; video‑coded eating rate; appetite VAS	Monteiro et al. 2018 (NOVA)^65^; texture varied independently	Texture was primary driver: hard meals ↓ intake (-21–26%); soft UPF produced highest intake. Least energy consumed from HMP vs SUP ↓ ∼300 kcal.
Rego et al. ^16^ USA	Healthy adults	*N* = 27; 22 y; 63% F; BMI 24	Eucaloric UPF diet	2 × 14 days	Eucaloric 0% UPF diet; matched nutrients and energy density	Energy intake (post‑diet buffet); Weighed food intake	UPF 81% vs. 0% Monteiro et al. (NOVA)^65^; verified by dietitian‑curated menus	No overall effect; UPF ↑ energy intake in 18–21 y vs. 22–25 y (subgroup analysis).

*BMI* body mass index, kg/m^2^, *CHO* carbohydrate, *DLW* doubly labelled water, *DXA* dual‑energy X‑ray absorptiometry, *EE* energy expenditure, *HMP* hard minimally processed (food), *ITT* intention‑to‑treat, *MP(F)* minimally processed (food), *NOVA* food processing classification system, *SUP* soft minimally processed (food), *UPF* ultra‑processed food, *VAS* visual analogue scale.

All studies classified foods using the NOVA framework (Monteiro et al.)^3,65^. Where applicable, UPF classification was verified by ingredient list and food label review; detailed procedures are described in the original publications.

## How processing drives risk (proposed mechanisms)

### Energy density and eating rate

UPFs are often characterised by softer textures and disrupted food matrices, which reduce oral processing demands and permit rapid consumption before satiation signals fully register, with potential implications in modifying gut–brain signalling^18^. A controlled 2 × 2 crossover study in 50 healthy-weight participants independently manipulated texture (soft vs. hard) and processing category (UPF vs. MPF) across four ab libitum meals (each separated by >7 d) (Table 2), allowing effect of texture on eating rate to be isolated from processing^15^. Soft meals were consumed at fasted eating rates and led to higher ab libitum energy intake than hard-textured meals, irrespective of processing category. However, the greatest intake occurred with soft UPF meals compared with hard MPF meals, reflecting combined effects of weakened matrix structure and rapid oral breakdown^15^.

**Table 2:** Texture and processing characteristics of experimental meal conditions (soft vs hard; ultra‑processed (UPF) vs minimally processed (MP) foods).

Processing Level	Texture	Example foods (as described in trial recipes 15)	Key structural features
Ultra-processed	Soft UPF	Instant mashed potato, tempura fish bites, coleslaw with creamy dressing, tartar sauce, flavoured yoghurt drink	Highly disrupted or reconstituted matrix; emulsifiers and additives; low structural integrity; reduced chewing requirement
Ultra-processed	Hard UPF	Potato waffle fries, mixed vegetable crisps, processed grilled chicken breast, dried mango	Increased hardness from frying or dehydration despite UPF; high chewing requirement
Minimally processed	Hard MPF	Multigrain rice blend, fresh salad vegetables, baked chicken breast	Intact cellular structure; high chewing requirement
Minimally processed	Soft MPF	Homemade mashed potato, steamed vegetables, yoghurt with seeds, avocado‑based sauce	Reduced hardness through cooking or blending without ultra-processing; reduced chewing requirement

These findings align with longer-term ad libitum evidence in 41 healthy participants showing that faster eating rates on ultra-processed diets are sustained over time and directly mediate excess energy intake, independent of macronutrient matching^19^. Reduced chewing frequency per calorie is similarly associated with marked increases in daily energy intake (~814 kcal/day) and rapid short-term weight gain (+1.1 kg in 1 week) in crossover feeding trials involving 50 adults with overweight/obesity^14^, reinforcing eating rate as a key mechanistic pathway linking food form, texture, matrix structure, and overconsumption^20^.

### Protein leverage

Small reductions in dietary protein percentage (e.g. 14% vs. 15–16%) can trigger compensatory increases in fat and carbohydrate intake; elevating total energy intake in line with protein leverage hypothesis and excess intake observed in UPF vs MPF diets^11,21^. In contrast, protein enrichment of UPFs (30% vs. 13%, crossover RCT, *n* = 21 healthy/overweight, 54 h) only modestly attenuates intake and does not prevent overeating, suggesting that protein content alone is insufficient to counteract hyperphagia in ultra-processed diets^13^.

### Food matrix and additives

Industrial food processing commonly refines and reformulates foods in ways that increase availability and rapid absorption of macronutrients, while concurrently reducing the content of intact fibre and associated micronutrients within food matrix^3,4^. This may limit delivery of fermentable substrates to the distal gut and plausibly influence microbial metabolism and downstream satiety signalling, as discussed in a recent mechanistic syntheses of UPF-obesity associations review by Juul et al.^4^. However, current experimental evidence linking UPFs to altered satiety hormone responses is limited and inconclusive, with small crossover RCTs^11,14^ observing a reduction or unchanged fasting Peptide YY and post‑intervention Glucagon-like peptide-1, likely due to being underpowered by small sample size (*n* < 20) and constrained by fasting‑state measurements. Emulsifiers (e.g. carboxymethylcellulose, polysorbate-80) may also compromise gut barrier integrity and promote low-grade inflammation in experimental models, with human data emerging^10,22^.

### High-fructose corn syrup (HFCS) and free sugars

HFCS is a sweetener produced from corn starch through industrial processing, commonly available in two principal forms 42% or 55% fructose along with glucose and water. Its lower cost and extended shelf life have driven widespread use in US SSBs and processed foods as a sucrose substitute^23^, whereas in Australia and Europe, sucrose remains dominant added sugar, with HFCS playing a comparatively minor role^24–26^. Mechanistic studies suggest high fructose intake may promote hepatic de novo lipogenesis, dyslipidemia, and hepatic fat accumulation, and that blunted insulin and leptin response—relative to glucose- may reduce satiety and energy expenditure, predisposing individuals to weight gain and obesity^27^. However, often such studies involve hypercaloric conditions or supraphysiological intake levels^28^. Under isoenergetic conditions, systematic review and meta-analysis evidence shows little support for fructose or HFCS adversely affecting energy intake or cardiometabolic risk markers compared with glucose or sucrose^27^. Elevated cardiometabolic risk appears to be primarily driven by excess energy – particularly from SSBs—rather than fructose per se^29^.

### Trans fatty acids (TFAs)

TFAs (unsaturated fats with trans double bonds) are naturally present in small amounts in dairy and meat. TFAs raise LDL, lower HDL cholesterol, impair endothelial function and fatty acid metabolism, increase inflammation, and elevate risks of CVD and all-cause mortality; even intakes >1% energy increase CHD risk^30–32^. Industrialised TFAs (iTFAs) arise mainly from partially hydrogenated oils (PHOs) in margarine and many processed foods (e.g. pastries, fried fast foods, popcorn) and are among most robustly evidenced dietary contributors to cardiovascular disease risk^33^. In line with global public health guidance, the World Health Organisation recommends intake of TFAs (including iTFAs) to be limited to <1% of total energy^34^, with iTFAs elimination from foods supply as preferred strategy^33^. iTFAs are banned in many countries (Denmark, EU, US, UK, Canada), but not Australia^30^.

### Hyper-palatability and reward

Many UPFs are engineered with supernormal combinations of fat, refined carbohydrates, salt, and flavour additives in ways that enhance palatability beyond natural foods, promoting excess intake through heightened reward and even addiction-like responsiveness^4,35^. Common UPF macro-combinations include: fat and simple sugars (>20% kcal from each); carbohydrates and sodium (>40% kcal from carbohydrates and ≥0.20% sodium by weight); fat and sodium (>25% kcal from fat and ≥0.30% sodium by weight))^35^. Evidence on non-nutritive sweeteners and metabolic outcomes (obesity, weight gain, insulin sensitivity, glycaemic control, and gut permeability) is mixed and context-dependent^36^.

### Potential brain changes

Neuroimaging studies (magnetic resonance imaging) in adults have revealed several structural brain changes associated with high UPF consumption. For example, high UPF consumption is associated with lower volumes in specific brain regions, particularly within mesocorticolimbic network, which is implicated in reward processing and conflict monitoring^37^. Notably, reductions in the volumes of posterior cingulate cortex and left amygdala^37^. In individuals with obesity, these associations extend to left ventral putamen and dorsal frontal cortex^37^. Additionally, high UPF intake in middle-aged adults (findings from UK Biobank cohort) has been linked to extensive grey matter compromise, including reduced subcortical volumes with a right-hemispheric predominance, and widespread cortical deterioration in volume, thickness, and surface area^38^. These structural changes are significant as they are associated with increased risks of neurodegenerative diseases such as dementia and Parkinson’s disease.

### Endocrine disruption

Packaging/thermal processing may increase exposure to potential endocrine disruptors (phthalates, bisphenols, acrylamide); however, evidence is primarily derived from in vitro experiments, animal models, and observation studies of highly exposed populations, with causal pathways and relevance to habitual dietary exposure in humans remaining insufficiently established^4^.

### Diet quality

UPFs tend to displace nutrient-dense whole foods, resulting in diets that are often lower in protective compounds (phytochemicals predominantly from fruits and vegetables) and poorer in overall diet quality^39,40^. However, growing consensus emphasises importance of disaggregating UPFs by nutritional quality, formulation, and food matrix characteristics, rather than treating all UPFs as nutritionally equivalent^41^. Improving overall diet quality, for example through higher intakes of fruits, vegetables and fibre-rich foods, may attenuate some adverse metabolic effects associated with UPF consumption^42–44^.

From a systems perspective, this implies that public health strategies should extend beyond consumer behaviour to include incentives for industry to improve nutritional composition of processed foods and to reduce processing practices that accelerate energy extraction, absorption, and intake rate. At same time, it is important to recognise that in certain contexts, particularly in settings affected by food insecurity or undernutrition, UPFs can provide safe, affordable and shelf-stable sources of energy, and may be preferable to insufficient food availability.

## Challenges for assessment and policy

### Defining ultra-processing

NOVA (a name, not an acronym) classification system, developed by Professor Carlos Monteiro and colleagues, is the most widely adopted framework for categorising foods and beverages based on their extent and purpose of industrial processing and provides a definition for UPF^3^. It classifies foods into four groups: Group1 (unprocessed or minimally processed foods; vegetables, fruits, nuts, plain meat, eggs, and milk, altered only by processes such as drying, grindings, or pasteurisation, without addition of salt, sugar, or fats); Groups 2 and 3 span culinary ingredients (oils, salt, sugar) and simply processed foods (canned fish, cheese)^3^; Group 4/NOVA 4 (UPFs) industrially formulated using substances rarely used in home kitchens (HFCS, hydrogenated oils, hydrolysed proteins, maltodextrins) and additives (emulsifiers, flavours, sweeteners, colours)^3^. NOVA 4 is widely used but the term is broad; its inherent heterogeneity– grouping disparate foods such as breakfast cereals, packaged breads, plant-based meat alternatives, and confectionery under a single umbrella based on processing extent rather than nutritional composition—invites misclassification and debate, yet the framework remains practical for surveillance and policy^3,45^.

### Measuring intake

Free-living assessments often rely on self-report (such as Food frequency questionnaires, 24-h dietary recalls), with measurement error, residual confounding, and rapidly evolving food supplies means that food composition databases struggle to keep pace with new products, complicating classification. Additionally, the application of NOVA to such dietary surveys is often inconsistent with incomplete ingredient information forcing research to make assumptions that risk misclassification. Objective biomarkers/‘omics’ platforms and AI-assisted tools could strengthen epidemiology and trials^8,10,46,47^.

### Socioeconomic realities

UPFs are often cheaper and more accessible than MPFs (e.g., $106/week vs. $151/week for 2000 kcal/day in one comparison), so policies must avoid widening inequities^11^.

### Reductionism

Processing level and nutrient quality should be considered together, focusing solely on either risks oversimplification. Some UPFs (e.g. certain wholegrain breads, low-sugar yoghurts, tomato sauces, nut/bean spreads) can contribute to healthy patterns and affordability, and should be monitored and reformulated if harms emerge^48^.

## Implications for dietary guidelines

While 2025–2030 Dietary Guidelines Advisory Committee initially judged UPF evidence too limited for formal recommendations given definitional and exposure inconsistencies, re-emphasising pattern-based guidance limiting processed meats, saturated fat, and added sugars (including SSBs)^49^, the subsequently published 2025–2030 Dietary Guidelines represent a historic shift where for first time explicitly advising Americans to avoid highly processed packaged and preprepared foods and SSBs^50^, underpinned by a commissioned umbrella review identifying moderate-to-high certainty evidence across T2D, dementia, CVD, cancer and all-cause mortality^51^. Notably, this umbrella review relied on a single-database search (PubMed only), atypical for a review of this scope, and applied GRADE upgrades to high certainty for T2D, dementia, and depression, and moderate-certainty evidence for all-cause mortality, cancer, CVD and obesity based on exclusively observational evidence, where dose-response gradients and large effect sizes, the primary upgrade criteria, may reflect shared measurement methodology and overlapping cohort populations across included meta-analyses rather than truly independent replication^51^. Nevertheless, even with accepting these strengthened certainty ratings, the absence of formal NOVA-based threshold recommendations contributes to ongoing definitional heterogeneity.

Several countries already address UPFs explicitly or implicitly: Brazil advises avoiding UPFs^52^; Uruguay promotes fresh/minimally processed patterns^52^; Israel prioritises home-cooked minimally processed foods and limiting highly processed items^53^; France limits charcuterie/SSBs but cautions against relying solely on processing classifications^54,55^; Spain (AESAN, 2022) integrates healthy/sustainable advice^52^; the UK SACN maintains a cautious stance, emphasising nutrient-based limits on fat, salt, and sugar as UPF-outcome associations may overlap with existing advice^4,56^; Australian dietary guidelines are currently under review^57^.

Professional societies converge on pattern-based dietary recommendations: the American Heart Association (AHA) advises choosing MPFs over UPFs within heart-healthy diets and recommends replacing most UPFs with healthier options (e.g. vegetables, fruits, whole grains, beans, nuts, seeds, healthy oils, and lean proteins) for cardiometabolic health^41,48^. While AHA acknowledges labelling foods as ‘ultra-processed’ can be misleading when nutrient-dense items are also included (e.g. nut butters) and assessing risks from processing techniques beyond nutrient quality remains challenging, as high UPF diets typically have poor overall quality and high-quality diets rich in UPFs are rare^48^.

On specific risk components, industrial TFA (iTFA) elimination is a proven, high-impact action (e.g. Denmark’s TFA legislation reduced CVD mortality, promoting global action including WHO’s ‘REPLACE’ initiative and bans across Europe, and US PHO elimination) ^30,33,41^. Australian modelling indicates a national iTFA ban would be cost-effective and prevent thousands of IHD events, yet such bans are not legislated^58^.

Finally, broader health-and-sustainability syntheses (e.g. EAT–Lancet) support planet-forward, minimally processed dietary patterns to reduce premature mortality and food-system emissions^59^.

## Beyond individual choice: a systems approach

Evidence on UPFs demands a shift from personal responsibility to systemic change. Despite decades of policy approaches centred on personal responsibility (e.g. calorie counting, nutrition education campaigns), obesity rates continue to rise in the US, UK, and Australia, where UPFs supply 40–60% of energy intake^2,4^. This failure reflects a mismatch between intervention strategies and root cause: food environments, not knowledge, shape diets. UPFs are engineered for palatability and profit, while minimally processed foods cost more. This dynamic is not uniform globally; in low- and middle-income settings, and among food insecure populations, affordable, shelf-stable UPFS can provide reliable energy and micronutrient fortification where fresh whole foods are inaccessible, unsafe, or insufficient; context therefore matters when translating evidence into recommendations^60,61^. Meaningful progress requires structural solutions (pricing, marketing restrictions, procurement standards, and labelling) to make healthier choices accessible and affordable. Critically, such transitions carry real costs: preparing less-processed food demands time, equipment, culinary skills, and money, representing a form of structural privilege not equitably distributed across socioeconomic groups. Without aligned incentives for industry, governments, and households, large-scale shifts in the food environment will remain aspirational.

**Research Priorities and the Path to Action**

- *Disaggregate UPFs* to identify attributes driving risk (matrix softness, energy density, additive classes, or their interactions); ongoing trials (e.g. UPDATE) will help^12^.
- Develop *objective exposure biomarkers and harmonised trial protocols* to improve reproducibility and generalisability.
- *Reformulation*: incentivise industry to reduce harmful attributes (e.g. specific emulsifiers, added sugars, sodium, iTFAs) while preserving affordability and convenience. Importantly, blanked reduction of all UPFs risks creating nutritional gaps- some NOVA 4 foods (fortified breads and cereals, flavoured yoghurts, peanut butter) contribute meaningfully to dietary adequacy, particularly in vulnerable populations; reformulation strategies should therefore target the most harmful subcategories rather than category as a whole.
- *Pragmatic trials* at scale to evaluate real-world implementation and impacts on population health; behaviour change interventions aimed at reducing UPF intake may yield greatest benefit in those at greater risk/ higher UPF intake^62^.

**From evidence to action: a multi-pronged playbook**

- *Guidance*: integrate processing level alongside nutrient content; offer practical swaps and preparation tips for time- and budget-constrained households.
- *Procurement and pricing*: leverage schools/hospitals/public programmes to improve availability and affordability of MPFs; pair with front-of-pack labelling that includes processing cues.
- *Industry standards*: set targets for added sugars, sodium, and select emulsifier classes; create positive incentives for industry to reformulate products that reduce energy extraction rate (eg. preserve food matrix integrity, reduce softening, lower energy density) alongside restrictions on the most harmful categories; prioritise categories with strongest evidence of harm (processed meats, SSBs, TFAs)^8^.
- *Protect children*: implement comprehensive marketing restrictions for HFSS/UPF products across media, aligned with WHO 2023 guidance^63^.
- *Food literacy*: invest in community-co-designed cooking and food skills programmes, tailored to vulnerable populations. Equity must be central; programmes should be co-designed with, not delivered to, lower-income communities, and paired with economic support to make participation realistic.
- *Evaluate*: fund longer, pragmatic trials to test real-world roll-outs focusing on improving overall dietary patterns.

## Conclusion

Controlled feeding trials demonstrate that processing per se can drive excess calorie consumption and adverse metabolic responses. Observational studies consistently associate high UPF intake with obesity, cardiometabolic disease, and all-cause mortality across diverse populations. While important questions remain about the relative contributions of specific UPF attributes, additive classes, and food matrix disruption—and while some UPFs continue to serve important roles in dietary adequacy, particularly in resource-limited settings—the weight of evidence is sufficient to act. The recent 2025–2030 US Dietary Guidelines signal that policy is beginning to reflect this reality. The question is no longer whether UPFs harm health, but how policymakers will confront commercial pressures, address structural inequities in food access, and redesign food systems so that minimally processed options become the default; affordable, accessible, and convenient for all. Integrating processing considerations into dietary guidelines alongside nutrient-based recommendations, paired with industry incentives to reformulate rather than simply restrict, offers a pragmatic and equitable path forward.

## Data Availability

No datasets were generated or analysed during the current study.
